# The first validated HPLC method with UV detection for concurrent assay of lidocaine and fluorescein in their co-formulated ophthalmic solution

**DOI:** 10.1186/s13065-026-01733-0

**Published:** 2026-02-19

**Authors:** Safa M. Megahed, Mahmoud M. Elshahawy

**Affiliations:** 1 Department of Pharmaceutical Analytical Chemistry, Faculty of Pharmacy, Tanta University, Tanta, Egypt; 2 Pharmaceutical Services Center, Faculty of Pharmacy, Tanta University, Tanta, Egypt

**Keywords:** Lidocaine, Fluorescein, HPLC, Eye drops, Quality control

## Abstract

A simple, fast, and eco-friendly high-performance liquid chromatography (RP-HPLC) method has been developed for the concurrent quantification of lidocaine and fluorescein, employing ultraviolet detection at a wavelength of 220 nm. The chromatographic separation was applied under isocratic conditions on an Inertsil^©^ C18 column (250 mm × 4.6 mm, 3.5 μm particle size), utilizing a mobile phase consisting of acetonitrile and water containing 0.3% triethylamine (adjusted to pH 3.5) in a volumetric ratio of 75:25%v/v. The analysis was performed at a constant flow rate of 1.0 mL/min. Method validation was conducted in accordance with the guidelines of International Council for Harmonisation (ICH), confirming that the procedure is accurate, precise, robust, and exhibits linearity within the concentration ranges of 20–280 µg mL^− 1^ for lidocaine and 1–20 µg mL^− 1^ for fluorescein. The detection limits were determined to be 5.27 µg mL^− 1^ for lidocaine and 0.29 µg mL^− 1^ for fluorescein, while the quantitation limits were 15.97 µg mL^− 1^ and 0.88 µg mL^− 1^, respectively. This analytical method was effectively applied to quantify both compounds in a combined ophthalmic formulation, yielding mean recovery values of 100.80 ± 0.67% for lidocaine and 100.13 ± 0.99% for fluorescein, thereby demonstrating its suitability for routine quality control purposes. The environmental impact of the proposed method was evaluated using Analytical Eco-Scale, GAPI, and AGREE tools, yielding an Eco-Scale score 78 and an AGREE score of 0.63, indicating acceptable greenness based on quantitative assessment.

**Supplementary Information:**

The online version contains supplementary material available at 10.1186/s13065-026-01733-0.

## Introduction

**Fig. 1:**
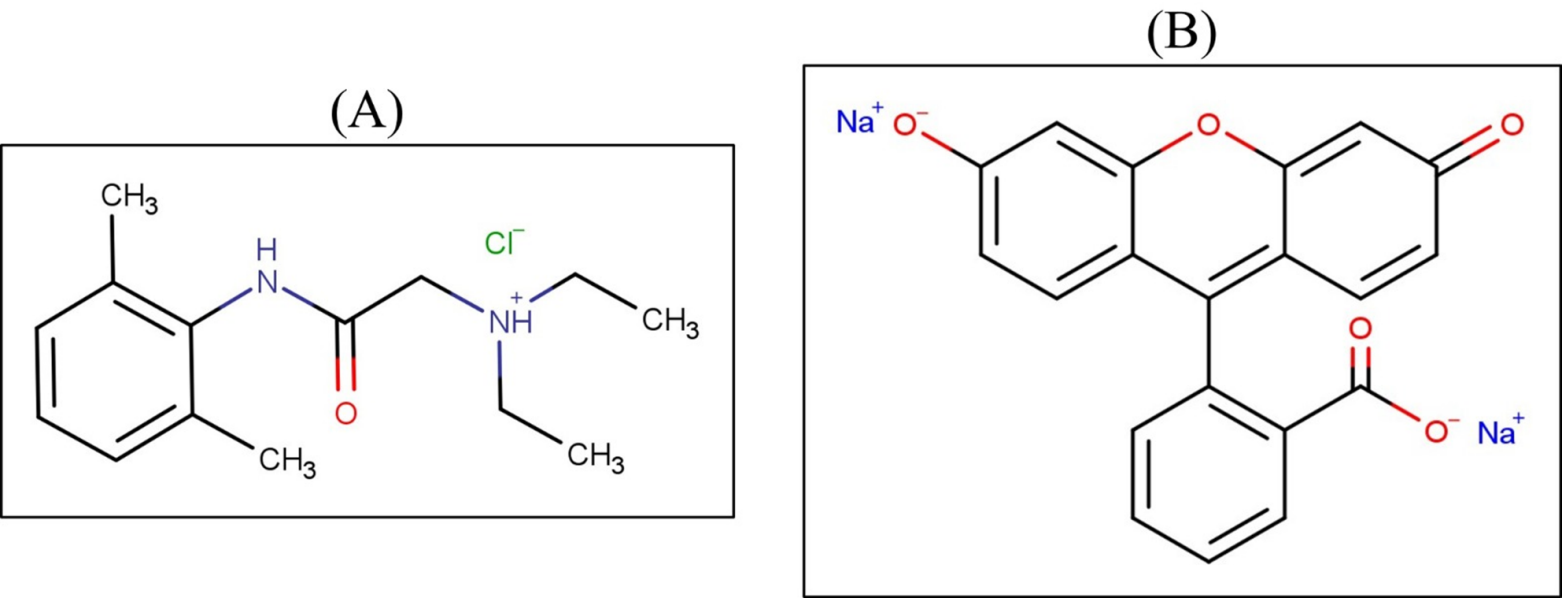
Chemical structures of **A** lidocaine and **B** fluorescein

Lidocaine hydrochloride, chemically identified as 2-(diethylamino)-N-(2, 6-dimethylphenyl) acetamide hydrochloride (Fig. 1A), is a local anesthetic of the amide type that functions by inhibiting the transmission of nerve impulses in the corneal sensory pathways. Fluorescein sodium, or disodium 2-(6-oxido-3-oxo-3 H-xanthen-9-yl) benzoate (Fig. 1B), is routinely employed in ophthalmic diagnostics to highlight corneal damage, such as abrasions, lesions, or the presence of foreign bodies. A combined ophthalmic preparation containing both lidocaine hydrochloride and fluorescein sodium is commonly utilized in procedures such as tonometry [1, 2]. Developing robust analytical methodologies for the concurrent quantification of these two agents in such formulations is essential to ensure product quality, therapeutic efficacy, and patient safety.

A variety of analytical methods have been found in the literature for the assay of lidocaine in pharmaceutical matrices. These include UV-Visible spectrophotometry [3, 4], fluorescence spectroscopy [5], liquid chromatography (HPLC) [6–10], gas chromatography [11], capillary electrophoresis (CE) [12], and electrochemical techniques [13–16]. Similarly, fluorescein has been quantified using methods such as UV-Visible spectrophotometry [17], fluorescence spectroscopy [18–20], HPLC [21–23], and capillary electrophoresis [24].

Although numerous sophisticated analytical methodologies have been published for the individual analysis of lidocaine and fluorescein, there appears to be a lack of reported methods addressing their simultaneous determination in co-formulated ophthalmic products. Given the combined use of these agents in such preparations, it is imperative to develop an analytical method that is both selective and efficient for their simultaneous quantification.

Green Analytical Chemistry (GAC) is increasingly discussed in the literature as a framework for reducing resource consumption in analytical procedures without compromising analytical reliability [25]. Recent studies illustrate that chromatographic methods can be optimized through shorter run times, reduced solvent volumes, and streamlined sample preparation, leading to lower waste generation while preserving separation efficiency [26–29]. Spectroscopic [30, 31] and fluorimetric [32, 33] techniques further contribute to this approach by exploiting high analytical sensitivity, which enables operation at low reagent and sample volumes. Electrochemical methods [34, 35] have also been reported as effective analytical alternatives due to their minimal solvent requirements and low energy demand. In this context, the use of quantitative evaluation tools such as the Analytical Eco-Scale, GAPI/ComplexGAPI, and AGREE has been proposed to objectively compare analytical methods based on resource use and operational parameters, rather than relying on qualitative or absolute environmental claims. The Eco-Scale evaluates methods by assigning penalty points for hazardous reagents, waste generation, and energy consumption, helping determine how environmentally friendly a procedure is [36]. GAPI offers a visual, step-by-step assessment using color-coded segments to show which parts of the workflow meet or fall short of green criteria [37]. AGREE summarizes the twelve principles of green analytical chemistry into a single score and circular diagram, providing an overall picture of method sustainability [38]. Together, these tools give a clear and comprehensive overview of an analytical method’s environmental performance.

This study focuses on the development and validation of ecofriendly HPLC method aimed at the concurrent determination of lidocaine and fluorescein in combined ophthalmic formulations. The proposed method is designed to be precise, accurate, and robust, capable of effectively separating and quantifying both analytes in the presence of one another. Its applicability for routine quality control testing of co-formulated ophthalmic solutions will also be demonstrated.

## Experimental

### Chemicals and materials

Fluorescein sodium (purity 99.20%) and lidocaine hydrochloride (purity 99.90%) were obtained from Sigma Pharmaceutical Industries (Quesna, Egypt). HPLC-grade acetonitrile was obtained from Fischer Scientific (Loughborough, UK). Triethylamine (TEA) was purchased from Sigma–Aldrich (Missouri, USA), while analytical-grade phosphoric acid was supplied by Riedel-de Haën (Weiden, Germany).

### High performance liquid chromatographic system

Chromatographic analyses were performed using a Dionex UltiMate-3000 RS system (Thermo Scientific™, Dionex™, USA), which consisted of a quaternary pump, an autosampler, and a photodiode array (PDA) detector. System control and data acquisition were managed using Chromeleon software, version 7.1. Separation was achieved on an Inertsil^©^C18 column (250 mm × 4.6 mm i.d., 3.5 μm particle size), supplied by GL Sciences Inc. (Tokyo, Japan). The mobile phase was a mixture of acetonitrile and an aqueous solution of 0.3% triethylamine, adjusted to pH 3.5 with phosphoric acid, in a volumetric ratio of 75:25% v/v. The flow rate was maintained at 1.0 mL/min, with the column temperature 25 °C. A 20 µL aliquot of each sample was injected, and detection was carried out using UV absorbance at 220 nm.

### Standard solutions

#### Stock standard solutions

A stock solution of lidocaine HCl at a concentration of 2000 µg mL^− 1^ was obtained by accurately weighing 200 mg of lidocaine and dissolving it in distilled water, followed by dilution to a final volume of 100 mL with the same solvent.

Similarly, a fluorescein sodium stock solution (500 µg mL^− 1^) was prepared by dissolving 25 mg of fluorescein in distilled water and adjusting the volume to 50 mL using the same diluent.

#### Working standard solutions

A working standard solution of lidocaine HCl at a concentration of 400 µg mL^− 1^ was prepared by transferring 50 mL of the previously prepared lidocaine HCl stock solution into a 250 mL volumetric flask then diluted to volume using distilled water.

To obtain a working fluorescein sodium solution with a concentration of 50 µg mL^− 1^, 10 mL of the fluorescein stock solution was transferred into a 100 mL flask, then completed to volume using distilled water.

A combined standard mixture of lidocaine HCl and fluorescein sodium was prepared by mixing 25 mL of the lidocaine HCl working solution with 12.5 mL of the fluorescein sodium working solution. The resulting mixture was diluted to a final volume of 50 mL with the mobile phase, yielding final concentrations of 200 µg mL^− 1^ for lidocaine and 12.5 µg mL^− 1^ for fluorescein.

### Laboratory-prepared ophthalmic solution

As a commercial ophthalmic formulation containing the combination of lidocaine HCl and fluorescein sodium is not currently available in the Egyptian pharmaceutical market, a simulated preparation was formulated in the laboratory to represent the intended dosage form. Precisely weighed quantities of 25 mg fluorescein sodium, 400 mg lidocaine HCl, and 1 g povidone were quantitatively transferred into a 10 mL volumetric flask. The contents were dissolved in distilled water, and the pH was adjusted to 7.4 using dilute hydrochloric acid. The final volume was brought up to 10 mL with distilled water, resulting in concentrations of 4% w/v for lidocaine HCl and 0.25% w/v for fluorescein sodium.

### Construction of lidocaine HCl calibration curve

Measured aliquots of the lidocaine HCl stock standard solution were transferred accurately into a number of 10- mL volumetric flasks. Each flask was then diluted to volume with the mobile phase to obtain a set of standard solutions with concentrations ranging from 20 to 280 µg mL^− 1^. Subsequently, 20 µL of each prepared solution was injected, in triplicate, into the chromatographic system. A calibration curve was established by plotting the average peak area against the corresponding lidocaine HCl concentrations.

### Construction of fluorescein sodium calibration curve

Accurate volumes of the fluorescein sodium stock standard solution were transferred into a number of 10- mL volumetric flasks, followed by dilution to the mark with the mobile phase to obtain standard solutions within the concentration range of 1 to 20 µg mL^− 1^. Each solution was injected into the chromatographic system in triplicate, using a 20 µL injection volume. The calibration curve was generated by plotting the average peak area against the corresponding concentrations of fluorescein sodium.

### Analysis of ophthalmic solution

A 250 µL aliquot of the laboratory-prepared ophthalmic formulation was transferred accurately into a 50- mL volumetric flask and completed to volume with the mobile phase, resulting in a solution containing 200 µg mL^− 1^ of lidocaine and 12.5 µg mL^− 1^ of fluorescein. The solution was filtered with a 0.45 μm syringe filter before HPLC injection. A 20 µL portion of this solution was injected, in triplicate, into the chromatographic system under the established conditions. The concentrations of lidocaine and fluorescein in the assay samples were determined using their respective calibration curves.

### Validation of the proposed method

#### Linearity and range

Calibration curves for lidocaine and fluorescein were constructed by plotting the average peak area against the corresponding analyte concentration, with each concentration level analyzed in triplicate. The linearity of the method was evaluated by regression statistics which include slope, intercept, standard error of slope, standard error of intercept, correlation coefficient and standard error of estimation.

#### Limits of detection (LOD) and quantitation (LOQ)

The limits of detection (LOD) and quantitation (LOQ) for the developed method were determined based on the standard deviation of the blank response (SD_blank_) and the calibration curve’s slope, using the following equations:

$$\:\mathrm{L}\mathrm{O}\mathrm{D}=\frac{3.3\:{\mathrm{S}\mathrm{D}}_{\mathrm{b}\mathrm{l}\mathrm{a}\mathrm{n}\mathrm{k}}}{\mathrm{s}\mathrm{l}\mathrm{o}\mathrm{p}\mathrm{e}}$$

$$\:\mathrm{L}\mathrm{O}\mathrm{Q}=\frac{10\:{\mathrm{S}\mathrm{D}}_{\mathrm{b}\mathrm{l}\mathrm{a}\mathrm{n}\mathrm{k}}}{\mathrm{s}\mathrm{l}\mathrm{o}\mathrm{p}\mathrm{e}}$$

#### Accuracy and precision

The accuracy of the developed method was assessed by analyzing three concentration levels of both lidocaine and fluorescein, selected within their respective linearity ranges. Each concentration was measured in triplicate, and the accuracy is expressed as mean percent recovery ± standard deviation.

Precision was evaluated through both repeatability and intermediate precision (intra-day & inter-day precision). For repeatability, three concentration levels were analyzed in triplicate within a single day. For intermediate precision, the same concentrations were tested over three consecutive days, with each level measured in triplicate daily. The precision is expressed as relative standard deviation (RSD).

#### Robustness

The robustness of the developed method was evaluated by introducing minor, intentional variations in some chromatographic parameters, including acetonitrile content (± 2%), pH (± 0.1 pH unit), flow rate (± 0.2 mL/min), and column temperature (± 2℃). The impact of these variations on the assay performance was assessed by calculating RSD for the percent recovery of lidocaine and fluorescein.

### Evaluation of method’s eco-friendliness

To quantitatively and qualitatively evaluate the environmental sustainability of the proposed analytical procedure, three complementary green analytical chemistry metrics were applied: the Analytical Eco-Scale, the Green Analytical Procedure Index (GAPI), and the Analytical GREEnness (AGREE) metric. The Analytical Eco-Scale provides a semi-quantitative assessment by assigning penalty points for deviations from an ideal green method, including factors such as the type and quantity of reagents, energy consumption, occupational hazards, and waste generation; these penalty points are subtracted from a base score of 100, with higher scores reflecting greater greenness and specific thresholds denoting excellent or acceptable green methods.

The GAPI tool complements this by offering a holistic visual evaluation of the entire analytical workflow through a set of color-coded pentagrams that represent key procedural stages, from sample collection and preparation to reagent use and instrumental operation, allowing rapid identification of environmentally significant steps.

Finally, the AGREE metric implements a comprehensive, principle-based quantitative assessment aligned with the 12 principles of Green Analytical Chemistry, standardizing each criterion on a 0–1 scale and integrating them into an interpretable overall greenness score via a dedicated calculator.

## Results and discussion

### Optimization of the chromatographic conditions

Various experimental parameters influencing the performance of the proposed method were systematically investigated and optimized. The primary objective of this optimization was to achieve well-resolved chromatographic peaks for both lidocaine and fluorescein, with minimal retention times and optimal peak symmetry, thereby enhancing the efficiency and reliability of the analytical procedure.

#### Selection of organic solvent

Ethanol, methanol, and acetonitrile were evaluated as organic modifiers in the mobile phase to assess their impact on chromatographic performance. Ethanol produced extended run times and broad, poorly defined peaks for both lidocaine and fluorescein. Methanol also resulted in prolonged analysis time and an excessively broad peak for fluorescein. In contrast, acetonitrile provided superior chromatographic characteristics, offering shorter run times and well-shaped, symmetrical peaks, and was therefore selected as the optimal organic solvent.

#### Type of aqueous medium

Several aqueous media were assessed to identify the most suitable component for the mobile phase, including acetate buffer, phosphate buffer, and TEA. Fluorescein failed to elute when acetate buffer was used, while the use of phosphate buffer led to broad peaks with noticeable tailing and shoulder formation. In contrast, TEA provided the most favorable performance, yielding sharp and symmetrical peaks for both analytes, and was thus selected as the optimal aqueous phase.

#### Effect of TEA concentration

Various concentrations of TEA, ranging from 0.1% to 0.5%, were systematically evaluated to determine their influence on peak tailing. At lower concentrations, pronounced tailing was observed. Increasing the TEA concentration led to progressive improvement in peak symmetry. The optimal concentration was found to be 0.3%, at which peak tailing was minimized. Further increases beyond this level did not yield any notable enhancement in peak shape, indicating that 0.3% TEA provided the most effective balance for optimal chromatographic performance.

#### Effect of pH

The pH of the aqueous TEA solution was adjusted using dilute phosphoric acid over a range of 3 to 7 to evaluate its effect on chromatographic behavior. Lidocaine, a weak base with a pKa of 7.75, and fluorescein, a weak acid with pKa values of 3.6 (carboxyl group) and 6.4 (phenolic hydroxyl group), exhibit pH-dependent ionization profiles [39]. Optimal resolution and minimal peak tailing were achieved at pH 3.5. At this pH, lidocaine’s tertiary amine is fully protonated, enhancing its polarity and leading to earlier elution. Meanwhile, the phenolic hydroxyl group of fluorescein remains largely unionized, resulting in a later elution with improved peak shape. As the pH increases, partial ionization of fluorescein’s phenolic hydroxyl group occurs, generating multiple ionic species. This results in peak splitting, increased tailing, and inversion of the elution order, ultimately compromising resolution. Therefore, pH 3.5 was identified as the optimal condition for achieving better separation with well-defined peaks.

#### Effect of acetonitrile ratio in the mobile phase

The effect of varying the proportion of acetonitrile in the mobile phase was systematically examined to optimize separation. At a high acetonitrile content of 90%, co-elution of lidocaine and fluorescein was observed, indicating inadequate resolution. Reducing the acetonitrile ratio gradually improved separation between the two analytes. At 70% acetonitrile, baseline resolution was achieved; however, the lidocaine peak exhibited shoulder formation. An acetonitrile content of 75% was identified as the optimal ratio, providing well-resolved, symmetrical peaks for both compounds with satisfactory chromatographic performance.

#### Effect of column temperature

The impact of column temperature on the chromatographic performance was evaluated over the range of 25 °C to 40 °C. It was observed that increasing the temperature led to a noticeable broadening of the lidocaine peak, adversely affecting peak shape and resolution. Consequently, a column temperature of 25 °C was determined to be optimal, ensuring sharper peaks and better separation efficiency.

**Fig. 2:**
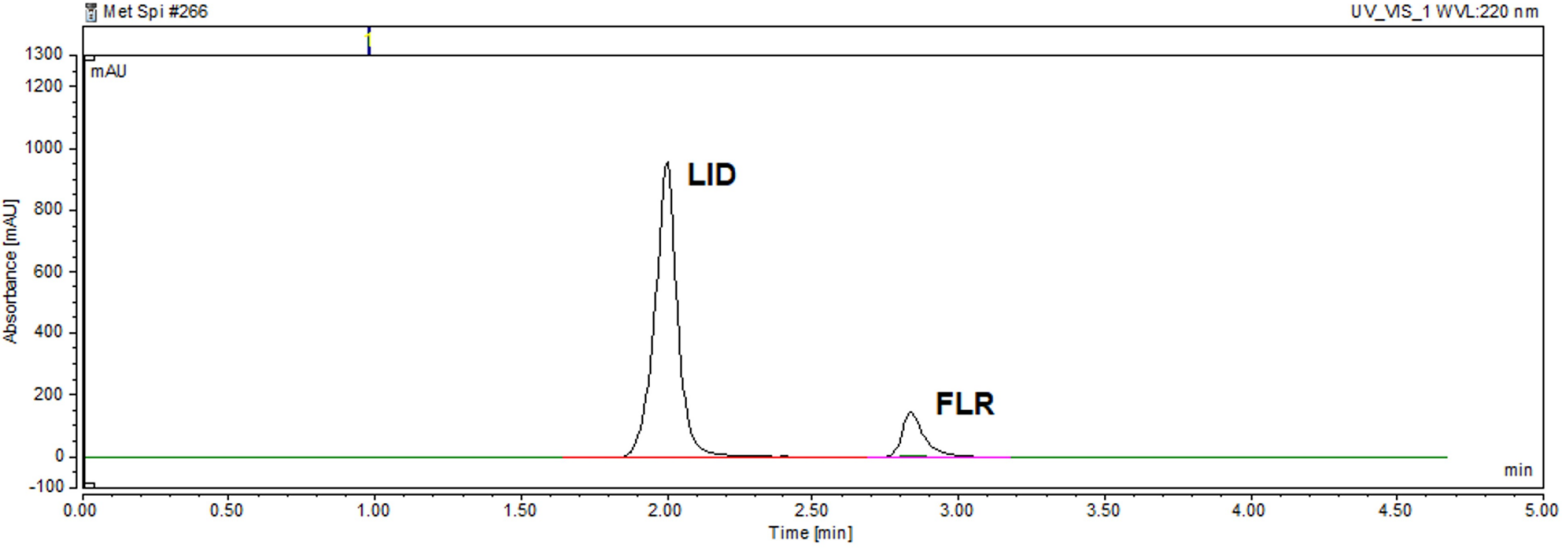
HPLC chromatogram of a mixture containing 200 µg mL^− 1^ of lidocaine and 12.5 µg mL^− 1^ of fluorescein at the optimum chromatographic conditions

The chromatogram of the lidocaine and fluorescein mixture obtained under these optimized conditions is presented in Fig. 2.

### Analytical performance and validation of the proposed method

The proposed analytical method was validated in accordance with the ICH Q2 (R1) guidelines [40]. The validation process encompassed the evaluation of key performance parameters, including linearity, analytical range, accuracy, limit of detection and quantitation (LOD & LOQ), precision, and robustness, to ensure the reliability and suitability of the developed method for routine analysis.

#### Linearity and range

Calibration curves for lidocaine and fluorescein were constructed by plotting the average peak area against the corresponding analyte concentration, with each concentration level analyzed in triplicate. The method demonstrated linearity within the concentration range of 20–280 µg mL^− 1^ for lidocaine and 1–20 µg mL^− 1^ for fluorescein. The regression statistics, presented in Table 1, confirm the adequacy of linearity for both compounds across their respective ranges, indicating the method’s suitability for quantitative analysis.

**Table 1:** Linearity regression parameters for Lidocaine HCl and fluorescein sodium using the proposed HPLC method

Parameter	Lidocaine HCl	Fluorescein sodium
Linearity range (µg/mL)	20–280	1–20
Slope	0.31	1.60
SE of slope	0.002	0.01
Intercept	1.08	0.17
SE of Intercept	0.37	0.12
Correlation coefficient (r)	0.9997	0.9997
SE of estimation	0.58	0.19

SE: Standard error

#### Limits of detection (LOD) and quantitation (LOQ)

The limits of detection (LOD) and quantitation (LOQ) for the developed method were determined based on the standard deviation of the blank response (SD_blank_) and the \ calibration curve’s slope.

Based on these calculations, the LOD values were 5.27 µg mL^− 1^ for lidocaine and 0.29 µg mL^− 1^ for fluorescein, while the LOQ values were 15.97 µg mL^− 1^ and 0.88 µg mL^− 1^, respectively. These results demonstrate that the developed method possesses sufficient sensitivity for the reliable quantification of both analytes in bulk materials, diluted samples, and pharmaceutical formulations.

#### Accuracy and precision

The accuracy of the developed method was assessed by analyzing three concentration levels of both lidocaine and fluorescein, selected within their respective linearity ranges. Each concentration was measured in triplicate, and the percent recovery was calculated. The results, presented in Table 2, are expressed as mean percent recovery ± standard deviation, confirming the method’s reliability in providing accurate quantification.

**Table 2:** Evaluation of accuracy for the determination of Lidocaine HCl and fluorescein sodium

Drug	Conc. taken (µg/mL)	Conc. found (µg/mL)	% Recovery	Mean % recovery ± SD
Lidocaine HCl	160	160.70	100.44	100.10 ± 0.46
	200	200.57	100.28	
	240	238.99	99.58	
Fluorescein sodium	10	9.97	99.86	99.61 ± 0.28
	12.5	12.41	99.31	
	15	14.95	99.66	

Precision was evaluated through both repeatability and intermediate precision (intra-day & inter-day precision). For repeatability, three concentration levels were analyzed in triplicate within a single day. For intermediate precision, the same concentrations were tested over three consecutive days, with each level measured in triplicate daily. As summarized in Table 3, the low relative standard deviation (RSD) values, each below 2.0%, indicate that the method exhibits high precision for the determination of both lidocaine and fluorescein.

**Table 3:** Evaluation of the precision of the proposed HPLC method for the determination of lidocaine HCl and fluorescein sodium

Drug	Intra day			Inter day		
	Conc. taken (µg/mL)	Conc. found (µg/mL)	%RSD	Conc. taken (µg/mL)	Conc. found (µg/mL)	%RSD
Lidocaine HCl	160	159.61	0.82	160	160.70	0.57
		160.33			160.65	
		162.17			162.33	
	200	199.73	0.37	200	200.57	0.16
		201.11			200.19	
		200.86			199.93	
	240	237.13	0.70	240	238.99	0.26
		240.32			238.97	
		239.53			240.07	
Fluorescein sodium	10	10.14	1.40	10	9.97	1.77
		10.04			10.17	
		9.87			10.32	
	12.5	12.73	1.76	12.5	12.56	1.59
		12.30			12.41	
		12.46			12.81	
	15	14.93	1.81	15	15.16	0.99
		14.60			14.95	
		15.13			15.24	

#### Robustness

The robustness of the developed method was evaluated by introducing minor, intentional variations in some chromatographic parameters, including acetonitrile content, pH, flow rate, and column temperature. The impact of these variations on the assay performance was assessed by monitoring the percent recovery of lidocaine and fluorescein. As summarized in Table 4, the method maintained consistent recoveries under all modified conditions, with relative standard deviation (RSD) values remaining below 2.0%. These results confirm the robustness of the method, indicating its reliability under slight fluctuations in experimental conditions.

**Table 4:** Robustness results for the proposed HPLC method

Parameters		Lidocaine HCl				Fluorescein sodium			
		Recovery %	Mean recovery %	S.D	R.S.D	Recovery %	Mean recovery %	S.D	R.S.D
% organic	78	99.17	99.91	0.52	0.52	101.14	100.91	0.50	0.49
	80	100.28				100.22			
	82	100.27				101.37			
pH	3.4	99.79	99.78	0.42	0.42	99.28	100.52	1.15	1.15
	3.5	100.28				100.22			
	3.6	99.27				102.06			
Flow rate	0.8	99.50	99.56	0.57	0.57	100.08	99.73	0.59	0.59
	1	100.28				100.22			
	1.2	98.88				98.90			
Temp	23	100.39	99.90	0.63	0.63	101.07	101.06	0.69	0.68
	25	100.28				100.22			
	27	99.01				101.90			

#### System suitability

System suitability of the chromatographic method was evaluated to ensure adequate performance prior to sample analysis. Parameters assessed included retention time, resolution, number of theoretical plates, and peak asymmetry factor. As summarized in Table 5, all measured values were within acceptable limits, confirming that the chromatographic system is suitable for the reliable and efficient separation and quantification of lidocaine and fluorescein.

**Table 5:** System suitability results for the proposed HPLC method

Parameters	Lidocaine HCl	Fluorescein sodium	Acceptance criteria**
Retention time (t_R_ min)*	1.97 ± 0.24	2.81 ± 0.41	–
capacity factor (k′)	1.56	2.43	1 ≤ k′≤5
Resolution (Rs)	6.65		Rs ≥ 2.0
HETP (cm)	0.066	0.029	–
Theoretical plates (N)	3811	8636	*N* ≥ 2000
Asymmetry factor	0.94	1.38	As ≤ 1.5

* Mean ± SD, *n* = 6

** According to USP

### Analysis of the laboratory-prepared ophthalmic solution

**Fig. 3:**
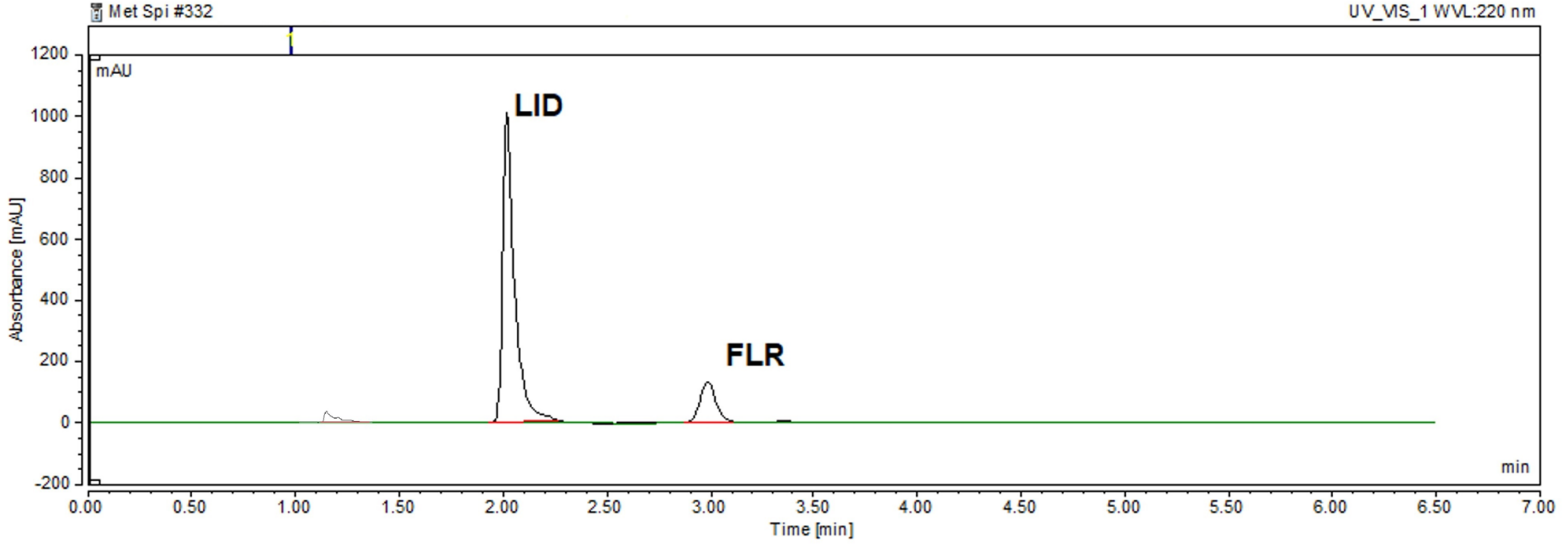
HPLC chromatogram of the laboratory-prepared ophthalmic solution containing 200 µg mL^− 1^ of lidocaine and 12.5 µg mL^− 1^ of fluorescein at the optimum chromatographic conditions

The developed HPLC method was successfully implemented for the concurrent quantification of lidocaine and fluorescein in the laboratory-prepared ophthalmic formulation. The chromatogram of the laboratory-prepared ophthalmic solution (Fig. 3) was comparable to that of the standard mixture containing equivalent concentrations of the two analytes (Fig. 2), indicating that the excipient, povidone, did not interfere with the detection or quantification of either drug. This absence of interference was further supported by recovery studies, in which six replicate analyses of the ophthalmic solution were performed. The mean percent recovery values, summarized in Table 6, confirm the method’s specificity and accuracy in the presence of formulation excipients.

**Table 6:** Application of the proposed method for the determination of the studied drugs in laboratory prepared eye drops

Drug	Concentration taken (µg/mL)	Mean concentration found * (µg/mL)	Mean % Recovery ± SD
Lidocaine HCl	200.00	201.59	100.80 ± 0.67
Fluorescein sodium	12.50	12.52	100.13 ± 0.99

* The mean of six determinations

SD: Standard deviation

###  Evaluation of chromatographic selectivity

The selectivity of the proposed HPLC method was evaluated to ensure reliable quantification of lidocaine hydrochloride and fluorescein sodium in the presence of formulation excipients. A placebo chromatogram, prepared using all excipients of the ophthalmic formulation without the active ingredients, was analyzed under the optimized chromatographic conditions. As shown in Fig S1, No peaks were observed at the retention times corresponding to either analyte, confirming the absence of excipient interference. In addition, UV spectra recorded across the leading edge, apex, and trailing edge of each analyte peak using PDA detection were superimposable, indicating peak homogeneity and absence of co-elution as shown in Fig S2. These findings demonstrate adequate chromatographic selectivity and confirm that the method is suitable for the analysis of the combined dosage form.

### Evaluation of method’s eco-friendliness

The sustainability characteristics of the proposed HPLC method were assessed using multiple complementary evaluation tools to provide a realistic and balanced appraisal rather than to claim full eco-friendliness. The Analytical Eco-Scale was applied to estimate the cumulative impact of solvent use, reagent hazards, energy consumption, and waste generation, and the obtained score (78) indicates acceptable sustainability relative to conventional chromatographic procedures as shown in Table S1. In addition, GAPI was used to visualize the environmental impact across the analytical workflow, including sample preparation, chromatographic separation, and waste handling. Furthermore, the AGREE metric was employed to evaluate the degree of alignment with the principles of Green Analytical Chemistry, yielding a moderate overall score that supports the method’s reasonable balance between analytical performance and solvent minimization.

**Fig. 4:**
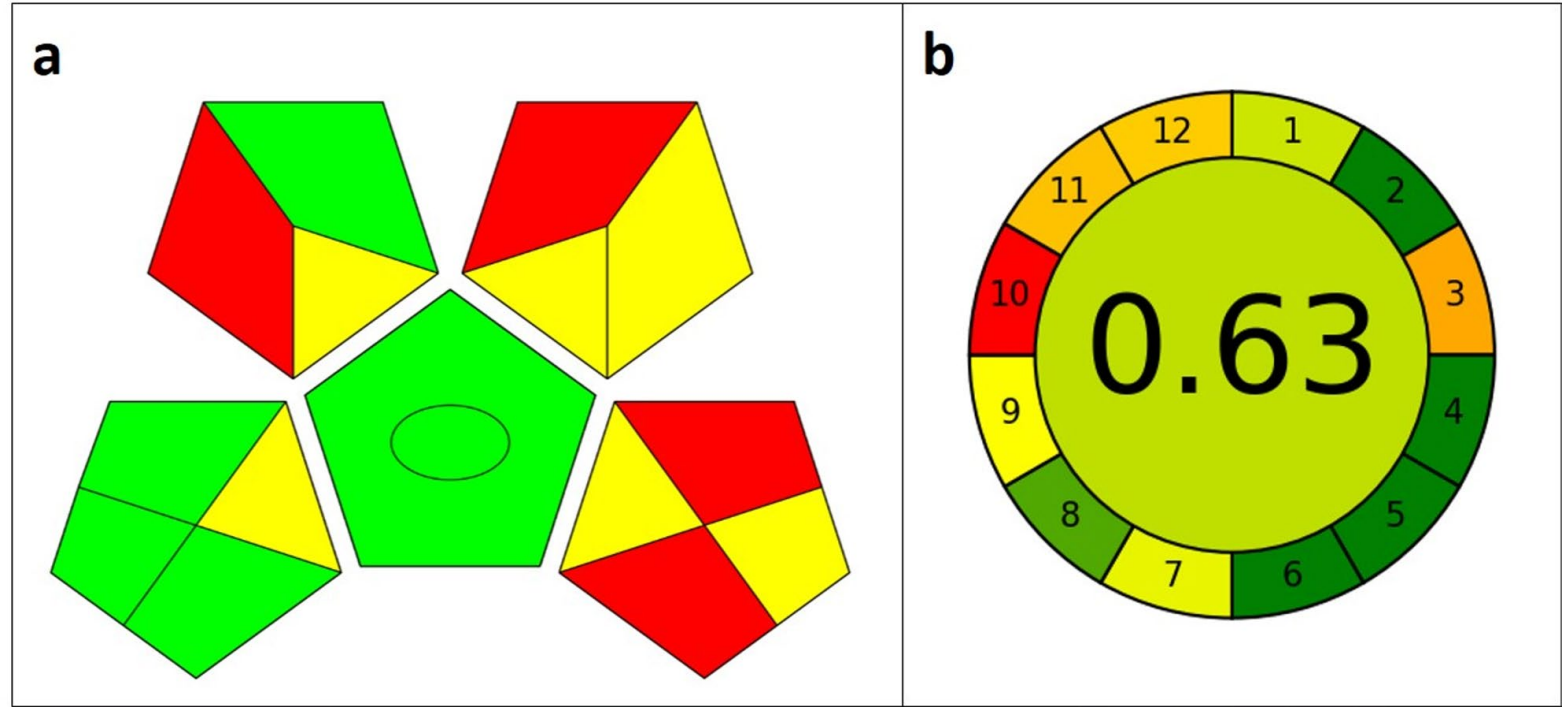
Evaluation of eco-friendliness of the developed method using **a** GAPI, and **b** AGREE tools

Overall, these assessments indicate that, although the method cannot be classified as fully eco-friendly, it incorporates measures that reduce solvent consumption and environmental impact compared with traditional HPLC approaches, while preserving robust analytical performance.The results of GAPI and AGREE evaluation are shown in Fig. 4.

## Conclusion

A novel eco-friendly RP-HPLC method has been successfully developed and validated for the concurrent quantification of lidocaine and fluorescein. This method is suitable for analyzing both drugs in their pure form as well as in co-formulated ophthalmic preparations. One of the prominent strengths of the proposed method lies in its simplicity, eliminating the need for complex sample preparation steps or advanced instrumentation, thereby making it accessible to a broad range of analytical laboratories. In addition to its straightforward implementation, the method offers a cost-effective solution for routine quality control, with rapid analysis achieved in a total run time of just 3 min. This contributes to enhanced operational efficiency, especially in high-throughput settings. The method underwent comprehensive validation in accordance with ICH guidelines, demonstrating excellent performance. Moreover, the absence of interference from formulation excipients, such as povidone, was confirmed through recovery studies and comparative chromatographic analyses, ensuring the specificity of the method. Overall, due to its speed, reliability, and ease of application, the developed HPLC method is highly suitable for routine quality control of lidocaine and fluorescein in pharmaceutical dosage forms, particularly ophthalmic solutions. The eco-friendliness of the developed method was proved using eco-scale, GAPI, and AGREE tools.

## Supplementary Information

Supplementary Material 1.

## Data Availability

All datasets generated and/or analyzed during the course of this study are provided within the main article and its supplementary materials. Additional data supporting the findings are available from the corresponding author upon reasonable request.

## Abbreviations

HPLC: High Performance Liquid Chromatography
TEA: Triethylamine
LOD: Limit of detection
LOQ: Limit of quantitation
RSD: Relative Standard Deviation

## References

[1] Brayfield A, editor. Martindale: the complete drug reference. 38th ed. London: Pharmaceutical; 2014.

[2] Moffat AC, Osselton MD, Widdop B, Watts J. Clarke’s Analysis of Drugs and Poisons. 4th ed. London: Pharmaceutical Press; 2011.

[3] Němcová I, Rychlovský P, Tomankova V, Živanovič L. Extraction spectrophotometric determination of Lidocaine using flow injection analysis. Anal Lett. 2001;34:2457–64.

[4] Omer LS, Ali RJ. Extraction-spectrophotometric determination of Lidocaine hydrochloride in pharmaceuticals. Int J Chem. 2017;9:1–49.

[5] Zhang Y, Gao Z, Zhang W, Wang W, Chang J, Kai J. Fluorescent carbon dots as nanoprobe for determination of Lidocaine hydrochloride. Sens Actuators B Chem. 2018;262:928–37.

[6] Al Nebaihi HM, Primrose M, Green JS, Brocks DR. A high-performance liquid chromatography assay method for the determination of lidocaine in human serum. Pharmaceutics. 2017;9:52. 29156554 10.3390/pharmaceutics9040052 PMC5750658

[7] Swezey CB, Ponzo JL. Liquid chromatographic determination of Lidocaine in serum. Clin Biochem. 1984;17:230–2. 6478584 10.1016/s0009-9120(84)90112-7

[8] Abdel-Rehim M, Bielenstein M, Askemark Y, Tyrefors N, Arvidsson T. High-performance liquid chromatography–tandem electrospray mass spectrometry for the determination of Lidocaine and its metabolites in human plasma and urine. J Chromatogr B Biomed Sci Appl. 2000;741:175–88. 10872587 10.1016/s0378-4347(00)00054-2

[9] Maes A, Weiland L, Sandersen C, Gasthuys F, De Backer P, Croubels S. Determination of lidocaine and its two N-desethylated metabolites in dog and horse plasma by HPLC–ESI–MS/MS. J Chromatogr B. 2007;852:180–7. 10.1016/j.jchromb.2007.01.010 17296334

[10] Habib AA, Mabrouk MM, Hammad SF, Megahed SM. Implementation of factorial design for optimization of forced degradation conditions and development of validated stability indicating RP-HPLC method for Lidocaine hydrochloride. Der Pharma Chem. 2015;7:198–211.

[11] Koster EHM, Wemes C, Morsink JB, De Jong GJ. Determination of lidocaine in plasma by direct solid-phase microextraction combined with gas chromatography. J Chromatogr B Biomed Sci Appl. 2000;739:175–82. 10744325 10.1016/s0378-4347(99)00344-8

[12] Valese AC, Spudeit DA, Dolzan MD, Bretanha LC, Vitali L, Micke GA. High-throughput analysis of Lidocaine in pharmaceutical formulation by capillary zone electrophoresis using multiple injections in a single run. J Anal Methods Chem. 2016;2016:4126810. 27069712 10.1155/2016/4126810 PMC4812394

[13] Oliveira RTS, Salazar-Banda GR, Ferreira VS, Oliveira SC, Avaca LA. Electroanalytical determination of Lidocaine in pharmaceutical preparations using boron-doped diamond electrodes. Electroanalysis. 2007;19:1189–94.

[14] Yang G, Zhao F. A novel electrochemical sensor for the determination of Lidocaine based on surface-imprinting on porous three-dimensional film. J Mater Chem C. 2014;2:10201–8.

[15] Saad AS, Al Alamein AMA, Galal MM, Zaazaa HE. Novel green potentiometric method for the determination of Lidocaine hydrochloride and its metabolite 2,6-dimethylaniline; application to pharmaceutical dosage form and milk. Electroanalysis. 2018;30:1689–95.

[16] Saad AS, Abou Al-Alamein AM, Galal MM, Zaazaa HE. Voltammetric determination of Lidocaine and its toxic metabolite in pharmaceutical formulation and milk using carbon paste electrode modified with C18 silica. J Electrochem Soc. 2019;166:B103.

[17] Mota MC, Carvalho P, Ramalho J, Leite E. Spectrophotometric analysis of sodium fluorescein aqueous solutions: determination of molar absorption coefficient. Int Ophthalmol. 1991;15:321–6. 1743867 10.1007/BF00128951

[18] Liu Y, Li YF, Huang CZ. Fluorimetric determination of fluorescein at the femtomole level with a self-ordered ring of a sessile droplet on glass slide support. J Anal Chem. 2006;61:647–53.

[19] Chen S, Nakamura H, Tamura Z. Determination of fluorescein and fluorescein monoglucuronide excreted in urine. Chem Pharm Bull. 1980;28:2812–6. 10.1248/cpb.28.2812 7460105

[20] Blair NP, Evans MA, Lesar TS, Zeimer RC. Fluorescein and fluorescein glucuronide pharmacokinetics after intravenous injection. Invest Ophthalmol Vis Sci. 1986;27:1107–14. 3721789

[21] Chen Y, Wang Y, Zhu M, Quan X, Shu J. HPLC determination of fluorescein sodium injection and its related substances. Chin J Pharm Anal. 2008;28:961–3.

[22] James IC, Lazo-Portugal R, Perez-Gonzalez M, Weisz A. Determination of fluorescein and brominated fluoresceins in the color additive D&c orange 5 and its lakes using HPLC. Food Addit Contam Part A. 2019;36:1800–7. 10.1080/19440049.2019.1664773 31535927

[23] Kamal AH, Habib AA, Hammad SF, Megahed SM. Quality by design paradigm for optimization of green stability indicating HPLC method for concomitant determination of fluorescein and benoxinate. Sci Rep. 2023;13:10471. 37380783 10.1038/s41598-023-37548-5 PMC10307890

[24] Ferguson PL, Grange AH, Brumley WC, Donnelly JR, Farley JW. Capillary electrophoresis/laser-induced fluorescence detection of fluorescein as a groundwater migration tracer. Electrophoresis. 1998;19:2252–6. 9761212 10.1002/elps.1150191234

[25] Keith LH, Gron LU, Young JL. Green analytical methodologies. Chem Rev. 2007;107:2695–708. 17521200 10.1021/cr068359e

[26] Sharaf YA, Abd El-Fattah MH, El-Sayed HM, Hassan SA. A solvent-free HPLC method for the simultaneous determination of favipiravir and its hydrolytic degradation product. Sci Rep. 2023;13:18512. 37898682 10.1038/s41598-023-45618-x PMC10613211

[27] Gohel J, Patel A, Kotadiya R. Green UHPLC approach for the quantitative determination of Tiopronin residues in cleaning validation processes. BMC Chem. 2025;19:1–13. 41137134 10.1186/s13065-025-01656-2 PMC12553291

[28] Hammad SF, Habib AA, Kamal AH, Megahed SM. Design of experiment-oriented development of solvent-free mixed micellar chromatographic method for concomitant determination of metronidazole and ciprofloxacin hydrochloride. Sci Rep. 2023;13:17352. 37833422 10.1038/s41598-023-44498-5 PMC10575926

[29] Megahed SM, Habib AA, Hammad SF, Kamal AH. Novel experimental design paradigm for development of eco-friendly gradient chromatographic method for simultaneous determination of metronidazole and spiramycin. J Sep Sci. 2023;46:2300216. 10.1002/jssc.202300216 PMC13583533 37654046

[30] ElDin NB, Abd El-Rahman MK, Zaazaa HE, Moustafa AA, Hassan SA. Supramolecular green chemistry: an eco-friendly spectrophotometric approach for determination of non-chromophoric methacholine via host–guest interactions with 4-sulfocalix[4]arene. Microchem J. 2021;168:106419.

[31] Madbouly EA, El-Shanawani AA, El-Adl SM, Abdelkhalek AS. Selective six spectrophotometric methods for determination of remdesivir and moxifloxacin hydrochloride for COVID-19 treatment with overlapping spectra: a comprehensive evaluation of greenness, blueness, and whiteness. BMC Chem. 2025;19:246. 40842025 10.1186/s13065-025-01607-x PMC12372283

[32] Hassan SAE, Ahmed SAEF, Helmy AH, Youssef NF. Spectrofluorimetric study on fluorescence quenching of tyrosine and L-tryptophan by the Aniracetam cognition enhancer drug: quenching mechanism using Stern–Volmer and double-log plots. Luminescence. 2020;35:728–37. 31994341 10.1002/bio.3778

[33] Abdelaal SH, El Azab NF, Hassan SA, El-Kosasy AM. Quality control of dietary supplements: an economic green spectrofluorimetric assay of raspberry ketone and its application to weight variation testing. Spectrochim Acta A Mol Biomol Spectrosc. 2021;261:120032. 34111836 10.1016/j.saa.2021.120032

[34] Farrag M, El-Mosallamy SS, Mohammed BS, Ahmed HM. Ultrasensitive green molecularly imprinted poly(o-phenylenediamine) sensor on pencil graphite for trace ertugliflozin quantification in plasma and tablets. BMC Chem. 2025;19:317. 41318490 10.1186/s13065-025-01681-1 PMC12670783

[35] ElDin NB, Abd El-Rahman MK, Zaazaa HE, Moustafa AA, Hassan SA. An eco-friendly potentiometric sensor for in-line monitoring of methacholine chloride hydrolysis: a “Green-Dip” approach. J Electroanal Chem. 2025. 10.1016/j.jelechem.2025.119371.

[36] Gałuszka A, Migaszewski ZM, Konieczka P, Namieśnik J. Analytical eco-scale for assessing the greenness of analytical procedures. TrAC Trends Anal Chem. 2012;37:61–72.

[37] Płotka-Wasylka J. A new tool for the evaluation of the analytical procedure: green analytical procedure index. Talanta. 2018;181:204–9. 29426502 10.1016/j.talanta.2018.01.013

[38] Pena-Pereira F, Wojnowski W, Tobiszewski M. AGREE—Analytical greenness metric approach and software. Anal Chem. 2020;92:10076–82. 32538619 10.1021/acs.analchem.0c01887 PMC7588019

[39] ChemAxon. MarvinSketch 22.12.0–1538. Budapest: ChemAxon Inc.; 2022.

[40] ICH. Q2 (R1.): Validation of analytical procedures: text and methodology. Geneva: International Conference on Harmonization. 2005.

