# Supplementary material for: The first validated HPLC method with UV detection for concurrent assay of lidocaine and fluorescein in their co-formulated ophthalmic solution
Source: BMC Chem. 2026 Feb 19;20(1):43. doi: 10.1186/s13065-026-01733-0 (PMC12930698; doi:10.1186/s13065-026-01733-0)
Supplement: Supplementary file 1 — Supplementary Material 1. [file 13065_2026_1733_MOESM1_ESM.docx]

**The First Validated HPLC method with UV Detection for Concurrent Assay of Lidocaine and Fluorescein in Their Co-Formulated Ophthalmic Solution**

Safa M. Megahed^1*^, Mahmoud M. Elshahawy^1,2^

^1^Department of Pharmaceutical Analytical Chemistry, Faculty of Pharmacy, Tanta University, Tanta, Egypt; ^2^Pharmaceutical Services Center, Faculty of Pharmacy, Tanta University, Tanta, Egypt

* Corresponding author: Safa Megahed

e-mail: [safa.megahed@pharm.tanta.edu.eg](mailto:safa.megahed@pharm.tanta.edu.eg), safa.megahed@gmail.com

Tel.: (+2)01004207905
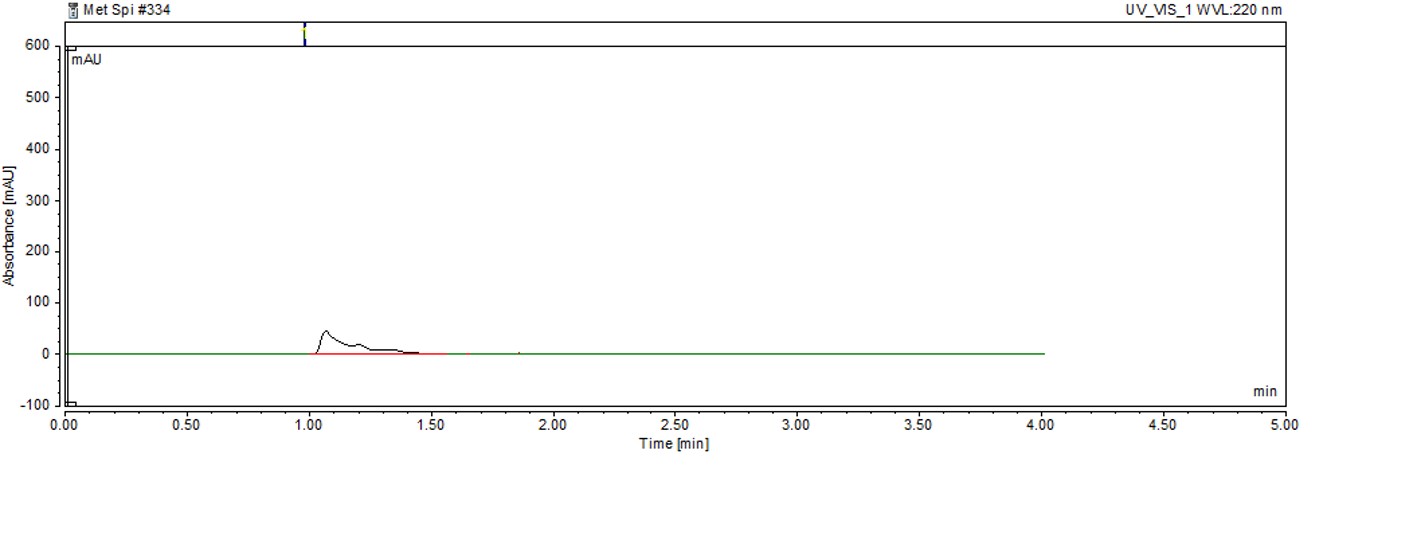


**Fig. S1:** Placebo chromatogram showing no interference from excipients.

**
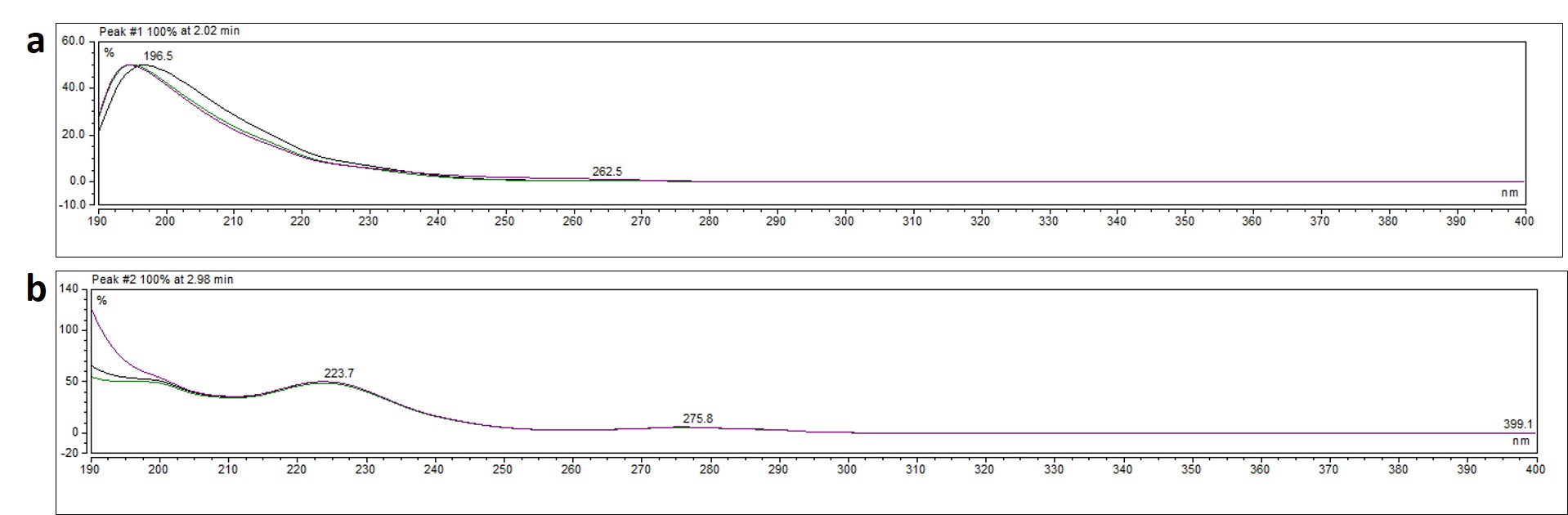
Fig. S2:** Overlaid UV spectra recorded at the leading edge, apex, and trailing edge of the chromatographic peaks of (a) lidocaine, and (b) fluorescein, demonstrating spectral homogeneity and confirming peak purity.

**Table S1:** Calculation of eco-scale for the proposed method

| **Reagents/Instruments** | **Penalty points** |
| --- | --- |
| **Reagents** | |
| Acetonitrile | 6 |
| Triethylamine | 6 |
| **Instruments** | |
| HPLC/UV | 1 |
| Occupational hazards | 3 |
| **Waste** | |
| Amount | 3 |
| No treatment | 3 |
| **Total penalty points** | ∑ 22 |
| **Analytical eco-scale score** | 78 |
